# K_2_CO_3_-Impregnated Al/Si Aerogel Prepared by Ambient Pressure Drying for CO_2_ Capture: Synthesis, Characterization and Adsorption Characteristics

**DOI:** 10.3390/ma13173741

**Published:** 2020-08-24

**Authors:** Yanlin Wang, Baihe Guo, Jingnan Guo, Man Zhang, Hairui Yang, Yan Jin

**Affiliations:** 1 College of Electrical and Power Engineering, Taiyuan University of Technology, Taiyuan 030024, China; 13453122526@163.com (Y.W.); guobaihehappy@163.com (B.G.); gguojingnan@163.com (J.G.); 2 Department of Energy and Power Engineering, Tsinghua University, Beijing 100000, China; zhangman@mail.tsinghua.edu.cn (M.Z.); yhr@mail.tsinghua.edu.cn (H.Y.)

**Keywords:** Al/Si aerogel, surface modification, potassium-based adsorbent, CO_2_ adsorption performance, adsorption kinetics

## Abstract

A new potassium-based adsorbent for CO_2_ capture with Al aerogel used as support is proposed in this work. The adsorbents with different surface modifiers (tetraethyl orthosilicate (TEOS) and trimethyl chlorosilane (TMCS)) and different K_2_CO_3_ loadings (10%, 20%, 30% and 40%) were prepared by sol-gel and iso-volume impregnation processes with ambient pressure drying. The CO_2_ adsorption performance of the adsorbents were tested by a fixed-bed reactor, and their adsorption mechanisms were studied by scanning electron microscopy (SEM), Brunauer Emmett Teller (BET), X-ray diffraction (XRD), Fourier transform infrared (FT-IR) spectroscopy, and X-ray fluorescence spectrometry (XRF). Furthermore, the adsorption kinetics and the cycling performance were investigated. The results show that using TEOS to modify the wet gel can introduce SiO_2_ to increase the strength of the skeleton. On the basis of TEOS modification, TMCS can further modify –OH, thus effectively avoiding the destruction of aerogel structure during ambient drying and K_2_CO_3_ impregnation. In this work, the specific surface area and specific pore volume of Al aerogel modified by TEOS + TMCS are up to 635.32 cm^2^/g and 2.43 cm^3^/g, respectively. The aerogels without modification (Al-B), TEOS modification (Al/Si) and TEOS + TMCS modification (Al/Si-TMCS) showed the best CO_2_ adsorption performance at 20%, 30% and 30% K_2_CO_3_ loading, respectively. In particular, the CO_2_ adsorption capacity and K_2_CO_3_ utilization rate of Al/Si-TMCS-30K are as high as 2.36 mmol/g and 93.2% at 70 degrees Celsius (°C). Avrami’s fractional order kinetic model can well fit the CO_2_ adsorption process of potassium-based adsorbents. Al-B-20K has a higher apparent activation energy and a lower adsorption rate during the adsorption process. After 15 adsorption-regeneration cycles, Al/Si-TMCS-30K maintain a stable CO_2_ adsorption capacity and framework structure, while the microstructure of Al/Si-30K is destroyed, resulting in a decrease in its adsorption capacity by nearly 30%. This work provides key data for the application of Al aerogel in the field of potassium-based adsorbent for CO_2_ capture.

## 1. Introduction

Global climate change caused by excessive CO_2_ emissions has become an indisputable fact [1]. Coal-fired power plants are the largest and most concentrated fixed source of CO_2_, and their annual CO_2_ emissions account for about 40% of total anthropogenic emissions [2]. Therefore, it is important to develop CO_2_ emission reduction technologies applicable to coal-fired power plants.

Post-combustion capture is a relatively mature technology, which is suitable for the removal of CO_2_ in the flue gas of traditional coal-fired power plants [3]. At present, the CO_2_ capture technology based on the alcohol-amine solution after combustion has achieved large-scale industrial applications [4,5,6], but its problems of high energy consumption for regeneration, corrosion and amine volatilization cannot be ignored. In contrast, the solid adsorbent can effectively avoid these problems [7]. In recent years, studies on the adsorption characteristics of CO_2_ by porous materials such as activated carbon, molecular sieves, carbon nanotubes, and metal organic frameworks (MOFs) have been widely carried out [8,9,10,11]. Although some materials have exhibited a good CO_2_ adsorption performance under high pressure, the CO_2_ adsorption properties of these materials are not satisfactory under normal pressure condition especially in the presence of water vapor: poor selectivity, low adsorption capacity and slow adsorption kinetics.

It has been found that the adsorption performance of porous materials can be improved significantly by loading active components that can interact with CO_2_ strongly [12,13]. Amine-based solid adsorbents are one of the most widely studied adsorbents, and its high CO_2_ adsorption capacity is widely concerned [14,15]. However, the amine-based adsorbent has many drawbacks such as secondary pollution, loss of active components, and high cost of the adsorbent. In the flue gas environment of the flue downstream of the coal-fired power plant, taking into account the adsorption and regeneration conditions of the adsorbent, the use of relatively inexpensive K_2_CO_3_ as an active component to capture and separate CO_2_ after desulfurization is a potential emission reduction technology [16]. K_2_CO_3_ can achieve CO_2_ adsorption by carbonation reaction, and can be regenerated at a relatively low temperature (120–150 °C). The adsorption and regeneration reaction equations are shown in Equations (1) and (2) [17].

(1) $$K_{2}{\mathrm{CO}}_{3}{+\mathrm{CO}}_{2}{+H}_{2}O\to {2\mathrm{KHCO}}_{3}$$

(2) $${2\mathrm{KHCO}}_{3}\to K_{2}{\mathrm{CO}}_{3}{+\mathrm{CO}}_{2}{+H}_{2}O$$

Lee et al. [18,19,20,21] and Zhao et al. [22,23,24,25,26] conducted in-depth research on the CO_2_ adsorption characteristics of supported potassium-based adsorbents. They found that the activity of K_2_CO_3_ mainly depends on the characteristics of the supports.

Adsorption capacity is an important indicator to measure the CO_2_ adsorption performance of potassium-based adsorbent. It directly determines the size of the adsorption equipment required in practical industrial applications, and then determines the cost of the adsorption system. Table 1 summarizes the CO_2_ adsorption capacity of potassium-based adsorbents with different supports [26,27,28,29,30,31,32,33,34,35], which are relatively low compared to amine-based adsorbents. Due to the limited loading capacity of the support to K_2_CO_3_, a too high loading will cause the microstructure of the adsorbent to deteriorate, which in turn affects the carbonation activity of the adsorbent [24,25]. Thus, the adsorbent needs to work at a lower loading. The lower loading leads to insufficient CO_2_ adsorption capacity of the adsorbent, which is an important factor restricting the adsorption performance of the potassium-based adsorbent.

It is found that the loading characteristics of K_2_CO_3_ mainly depend on the microscopic characteristics of the support [36], therefore, it is a meaningful work to find the materials with high load capacity for K_2_CO_3_. Aerogel is a light porous condensed matter with air as the medium, it is a unique nanoporous three-dimensional network structure composed of colloidal particles or polymer molecules that aggregate with each other. It has a very high specific surface area and porosity, with adjustable open pore structure and an easily chemically modified surface, and has a wide range of applications in the field of adsorption [37,38]. At present, research has been widely carried out on SiO_2_ aerogel and carbon aerogel in the field of CO_2_ adsorption, and the active components are mostly amino groups [39,40,41]. Linneen et al. [41] prepared the SiO_2_-supported tetraethyl pentamine (TEPA) amino-functional CO_2_ adsorbent by impregnation method, the TEPA loading capacity can reach 80 wt.%, and the adsorption capacity can reach 6.1 mmol/g at 75 °C under pure CO_2_ atmosphere, however, due to the hydrophilicity of the SiO_2_ aerogel surface, the structure of the aerogel contracted during the impregnation process. Toufigh et al. [42] prepared K_2_CO_3_-based CO_2_ adsorbent based on Al_2_O_3_ aerogel prepared by supercritical drying. The aerogel was calcined at 1000 °C to remove surface –OH before impregnation, when the K_2_CO_3_ loading is 50%, the maximum CO_2_ adsorption capacity can reach 7.2 mmol CO_2_/g K_2_CO_3_.

Alumina is a potential support for CO_2_ adsorbent, and combining SiO_2_ and Al_2_O_3_ can give aerogel unique advantages. To the best of our knowledge, there are few reports on its research in the field of potassium-based CO_2_ adsorbents. At present, the preparation of aerogels by supercritical drying technology (SCD) has achieved commercial application, however, the technology is complicated, with poor safety and high cost. Therefore, the ambient pressure drying technology used for aerogels has attracted much attention [43], which usually reduces the shrinkage and collapse of the skeleton during the drying process through surface modification and other means during the formation of the wet gel. At the same time, in order to make the aerogel suitable for the impregnation process commonly used in K_2_CO_3_ load, surface hydrophobic modification treatment is required. The role of the hydrophobic modifier and its effect on CO_2_ adsorption performance are currently unclear.

In industrial applications, in addition to a high CO_2_ adsorption capacity, the adsorbent should have a fast adsorption rate, which is directly related to the efficiency of the CO_2_ capture process. Researchers have conducted in-depth research on the adsorption kinetics of amine-based adsorbents [44,45]. However, there are few studies on the adsorption kinetics of K_2_CO_3_/Al aerogels [46]. In addition, the stability of the adsorbent during the adsorption-regeneration cycles is also an important indicator in practical industrial applications, so it needs to be investigated.

In this work, potassium-based adsorbents with Al aerogels used as supports were prepared by sol-gel and iso-volume impregnation method. The effects of the microstructures of the supports and the loadings of K_2_CO_3_ on the adsorption performance and the mechanism were studied. Meanwhile, the pseudo-first order, pseudo-second order, and Avrami’s fractional order models were used to fit the adsorption data to study the adsorption rate and adsorption mechanism. Furthermore, the cycle stability of the adsorbents in the continuous adsorption-regeneration process was investigated.

## 2. Materials and Methods

### 2.1. Preparation of Adsorbents

**Figure 1:**
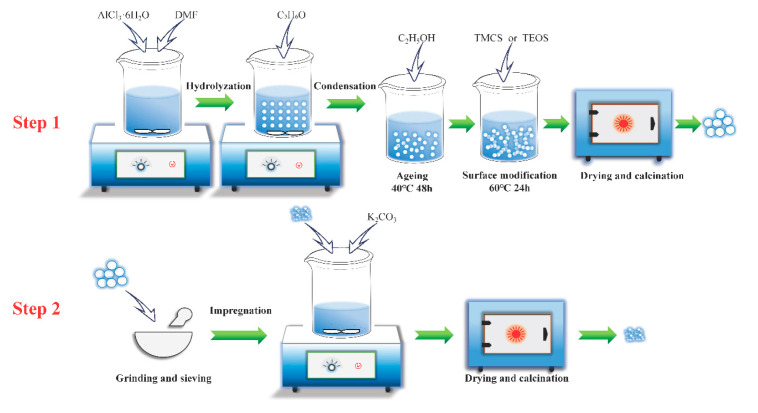
Schematic diagram of the preparation process of adsorbent.

Figure 1 is a schematic diagram of the preparation process of the adsorbents. The specific steps are as follows:

(1) A certain amount of AlCl_3_·6H_2_O (Tianjin Kaitong Chemical Reagent Co., Ltd., Tianjin, China) and *N*,*N*-dimethylformamide (DMF, Tianjin Kaitong Chemical Reagent Co., Ltd., Tianjin, China) were completely dissolved in deionized water; specifically, DMF can control the rate of hydrolysis-polycondensation to improve the uniformity of network channels. Then, propylene oxide (C_5_H_6_O, Tianjin Fangzheng Reagent Co., Ltd., Tianjin, China) was added to the solution under stirring; specifically, AlCl_3_·6H_2_O:H_2_O:DMF:C_5_H_6_O = 1:20:8:0.8 (molar ratio). After the wet gel was formed, it was aged for 48 h at 40 °C in ethanol solution (C_2_H_5_OH, Tianjin Kaitong Chemical Reagent Co., Ltd., Tianjin, China).

(2) Surface modification was performed on the aged wet gel. The modifiers were ethanol solution (blank control group), 80 wt.% tetraethyl orthosilicate (TEOS, Tianjin Fangzheng Reagent Co., Ltd., Tianjin, China) in ethanol solution (Modifier A) and 80 wt.% trimethyl chlorosilane (TMCS, Tianjin Fangzheng Reagent Co., Ltd., Tianjin, China) in ethanol solution (Modifier B), respectively. The modification schemes were: no modification, modifier A modification and modifier A and B modification sequentially, respectively. During modification, the wet gel was immersed in the modifier at 60 °C for 24 h.

(3) The modified wet gel was staged drying under ambient pressure, and then calcined at 600 °C for 3 h to obtain Al aerogel, the samples were recorded as Al-B, Al/Si, Al/Si-TMCS according to the modifiers.

(4) The iso-volume impregnation method was used to prepare potassium-based adsorbent. The particle size of the support was selected as 90–120 μm, and the design loading of K_2_CO_3_ (Nanjing Caiwei Technology Co., Ltd., Nanjing, China) was in the range of 0–40 wt.%. After drying and calcining, the adsorbent was re-sieved to the same size range as the support.

### 2.2. Experimental Apparatus and Procedure

**Figure 2:**
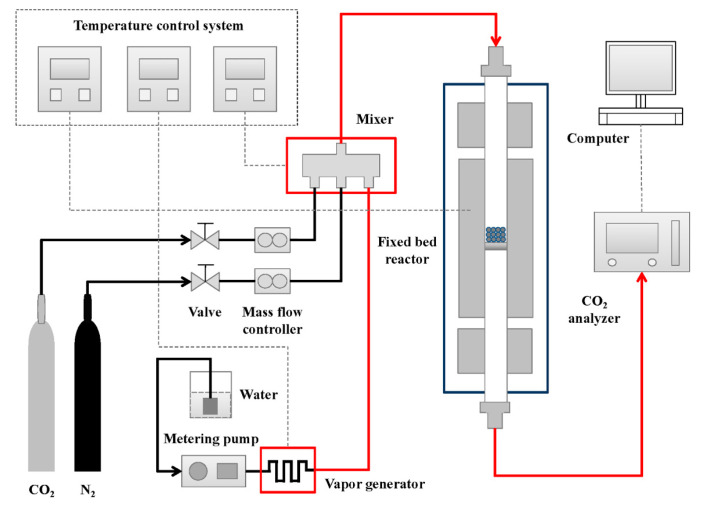
Fixed bed CO_2_ adsorption system.

The CO_2_ adsorption experiment was performed in a fixed-bed reaction experiment system. Figure 2 is a simplified diagram of the experimental system. In order to simulate the flue gas environment of the coal-fired power plant, the adsorption atmosphere was selected as 80% N_2_ + 10% CO_2_ + 10% H_2_O. H_2_O was controlled by Series III metering pump and was vaporized using electrical heating to the water vapor required for the carbonation reaction. The adsorption process was performed in a quartz tube with an inner diameter of 30 mm and a height of 300 mm, and 3 g adsorbent was placed in the quartz tube. The Xinze S2000 infrared CO_2_ detector (Shandong Xinze Technology Co., Ltd., Jinan, China) was used to measure the outlet CO_2_ concentration.

### 2.3. Data Process

The CO_2_ adsorption capacity and K_2_CO_3_ utilization rate were calculated by Equations (3) and (4), respectively.

(3) $$q\;=\;\frac{1000}{m}\left[\overset{t}{\underset{0}{\int }}Q_{a}\left(\frac{C_{\mathrm{in}}-C_{\mathrm{out}}}{1\;-{\;C}_{\mathrm{out}}}\right)\mathrm{dt}\right]\frac{T_{0}}{{\mathrm{TV}}_{m}}$$

(4) $$\eta \;=\;\frac{q\;-{\;q}_{p}}{\alpha /M_{K_{2}{\mathrm{CO}}_{3}}\times \;1000}$$

where q is the total CO_2_ adsorption capacity, mmol/g; q_p_ is the physical adsorption capacity, mmol/g; C_in_ is the inlet CO_2_ concentration, %; C_out_ is the outlet CO_2_ concentration, %; Q_a_ is the gas flow rate, mL/min; m is the mass of the adsorbent, g; t is the adsorption time, min; T is the adsorption temperature, K; T_0_ is the gas temperature in standard state, T_0_ = 273.15 K; V_m_ is the molar volume of gas in standard state, V_m_ = 22.4 L/mol; α is the actual loading, %; $M_{K_{2}{\mathrm{CO}}_{3}}$ is the relative molecular mass of K_2_CO_3_, $M_{K_{2}{\mathrm{CO}}_{3}}$ = 138.

### 2.4. Characterization Method

An N_2_ adsorption-desorption experiment of the adsorbent was performed by ASAP2420 physical adsorption-desorption instrument (Micromeritics Instrument Co., Ltd., Norcross, GA, USA), the specific surface area was calculated by the BET equation, and the pore structure parameters were obtained by the BJH method; Tescan Mira3 scanning electron microscope (SEM, Tescan Co., Ltd., Brno, Czech Republic) was used to characterize the micro-morphology of the adsorbent; phase structure of the adsorbent was characterized by an X/max-2500 X-ray diffractometer (XRD, Rigaku Co., Ltd., Tokyo, Japan) with Cu Kα radiation at a scanning rate of 2°/min over the range of 2° < 2θ < 85°. The potassium content of the adsorbent was measured by ARL9800XP X-ray fluorescence spectrometer (XRF, ARL Co., Ltd., Kanton Bern, Switzerland); and the surface groups were analyzed by an IRPrestige-21 Fourier transform infrared (FT-IR, Daojin Co., Ltd., Hongkong, China) spectrometer.

## 3. Results and Discussion

### 3.1. Characterization

**Figure 3:**
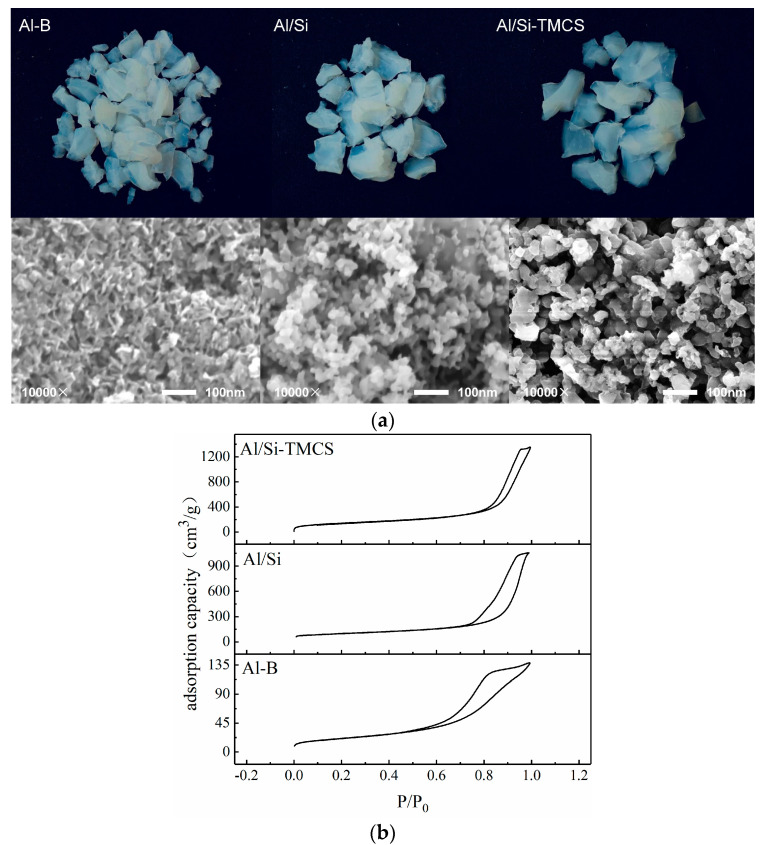

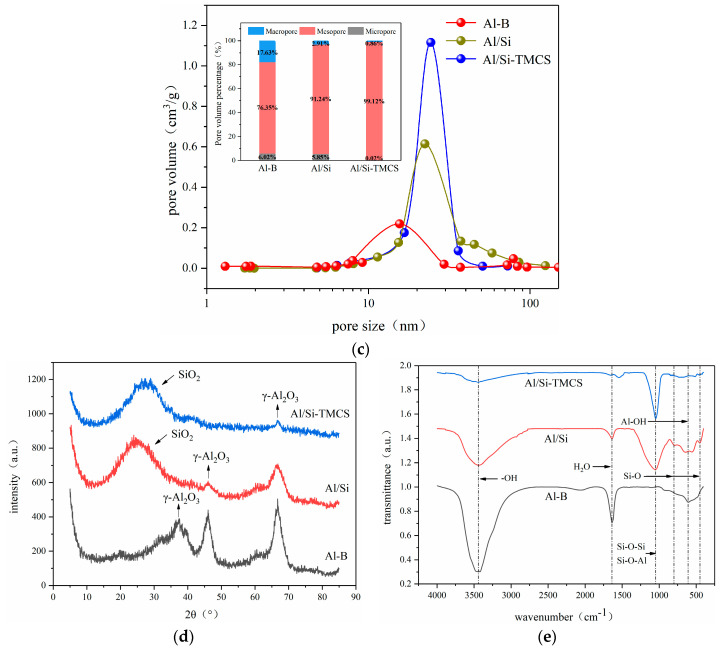
Characterization results of Al_2_O_3_ aerogels. (**a**) macroscopic and microscopic surface structures of three types of Al aerogels; (**b**) N_2_ adsorption-desorption curves of three types of Al aerogels; (**c**) Pore size distributions of three types of Al aerogels; (**d**) X-ray diffraction (XRD) spectra of three types of Al aerogels; (**e**) Fourier transform infrared (FT-IR) spectra of three types of Al aerogels.

Figure 3a shows the macroscopic and microscopic surface structures of Al-B, Al/Si and Al/Si-TMCS. The three types of aerogels are translucent solids, Al/Si and Al/Si-TMCS showed larger particles than Al-B, indicating that the ability to stay intact during drying is stronger, while Al-B is broken to a greater extent during ambient pressure drying. SEM results show that Al aerogels obtained under different preparation conditions are all sponge-like structures with relatively uniform pores, which are formed by stacking irregular nanoparticles. and Al-B has a smaller pore size.

Figure 3b,c show the N_2_ adsorption-desorption curves and pore size distributions of different samples. According to the classification of the International Union of Pure and Applied Chemistry (IUPAC), the N_2_ adsorption-desorption curves of the three adsorbents belong to the typical type IV isotherm. Meanwhile, Al-B has a typical H2 type hysteresis loop, which corresponds to an ink bottle-shaped pore, and the uniformity of the pore structure is relatively poor. Al/Si-TMCS has a typical H1-type hysteresis loop, and the adsorption and desorption branches are steep and almost parallel, indicating the presence of tubular capillary pore with well-organized shape and uniform size. And the hysteresis loop type of Al/Si is between the H1 type and H2 type. It can be seen from the pore size distribution that the pore sizes of the three samples are mainly mesoporous. In particular, the modification of TEOS and TMCS narrowed the pore size distributions of the aerogels, and the proportion of mesopores of Al/Si and Al/Si-TMCS reached more than 90%. A study has shown that a single pore size distribution on the sample surface can make the surface stress more uniform, thereby making it easier for the active component to disperse spontaneously [47]. Al-B has a relatively small pore size, and some micropores and macropores appear, indicating that network shrinkage and structural collapse occur during atmospheric drying and calcination.

The pore structure parameters of the three samples are listed in Table 2. According to Table 2, the maximum specific surface area (635.32 m^2^/g) and specific pore volume (2.43 cm^3^/g) belong to Al/Si-TMCS, and its specific surface area is higher than that of Al/Si aerogel prepared by Wu [48] using SCD technology (512.50 m^2^/g at 600 °C). The specific surface area and specific pore volume of Al-B without TEOS and TMCS modification are 250 m^2^/g and 0.49 cm^3^/g, respectively, which is much lower than that of Al/Si and Al/Si-TMCS, indicating that TEOS and TMCS play an important role in maintaining the structure of aerogel during ambient drying.

The XRD spectra of Al aerogels prepared with different modifiers are shown in Figure 3d. The presence of γ-Al_2_O_3_ is detected in all three samples (2θ = 37°, 46° and 66°), this is formed by the gradual dehydration during the calcination process of the aluminum hydroxide polymer produced by the hydrolysis-polycondensation of the inorganic precursor AlCl_3_·6H_2_O. Meanwhile, after being modified by TEOS, the XRD result of Al/Si shows a diffuse peak of amorphous SiO_2_ around 2θ = 25°, which indicates that SiO_2_ was formed due to the hydrolysis-polycondensation reaction of TEOS during the modification process, and a study has shown that the coating of SiO_2_ can strengthen the framework structure of Al_2_O_3_ aerogel [49]. The modification of TMCS does not have much of an effect on the phase composition of Al/Si aerogel. It is worth noting that γ-Al_2_O_3_ is more amorphous in Al/Si and Al/Si-TMCS compared to Al-B, which is caused by less –OH on the surface, literature [49] has shown that less –OH can inhibit the transformation of Al_2_O_3_ from amorphous to crystalline structure at high temperature.

Figure 3e shows the FT-IR spectra of three types of Al aerogels. The strong and wide peak at 3435 cm^−1^ belongs to the stretching vibration of surface-associated hydroxyl group (–OH), and the peak at 1630 cm^−1^ corresponds to the bending vibration of –OH in physically adsorbed H_2_O, and the absorption peaks at 882 cm^−1^ and 627 cm^−1^ correspond to Al–OH. –OH is a hydrophilic group, which are prone to occur polycondensation reaction to cause the gel structure to shrink during the drying process of wet gel. The modification of TEOS makes the surface –OH have a certain reduction, at the same time, the vibration of Si–O–Si and Si–O–Al groups appeared at 1095 cm^−1^, this is because TEOS can undergo a hydrolysis-polycondensation reaction to produce Si–O–Si groups, meanwhile, –OH on the surface of the polycondensation product may also be condensed with Al–OH on the surface of the wet gel to produce Al–O–Si [48]. On the basis of TEOS modification, TMCS can further modify the –OH on the surface of the wet gel, so that the peaks at 3435 cm^−1^ and 1630 cm^−1^ in Al/Si-TMCS almost disappear, which greatly reduces the hydrophilicity of aerogel.

### 3.2. Loading Characteristics

In order to increase the adsorption capacity and selectivity of Al aerogel for CO_2_, K_2_CO_3_ was loaded with iso-volume impregnation method. The design loadings are selected as 0%, 10%, 20%, 30% and 40%, and the prepared adsorbents were recorded as Al-B-0K, Al-B-10K, Al-B-20K, Al-B-30K, Al-B-40K, Al/Si-0K, Al/Si-10K, Al/Si-20K, Al/Si-30K, Al/Si-40K, Al/Si-TMCS-0K, Al/Si-TMCS-10K, Al/Si-TMCS-20K, Al/Si-TMCS-30K, Al/Si-TMCS-40K. Among them, Al-B-0K, Al/Si-0K and Al/Si-TMCS-0K were used as blank experiments to test the effect of the impregnation process on the structure of the sample. At the same time, they were used to test the water absorption of the support to determine the volume of the K_2_CO_3_ impregnating solution during the impregnation. The microstructure parameters measured by N_2_ adsorption-desorption experiment and the actual K_2_CO_3_ loading measured by XRF are listed in Table 3.

**Figure 4:**
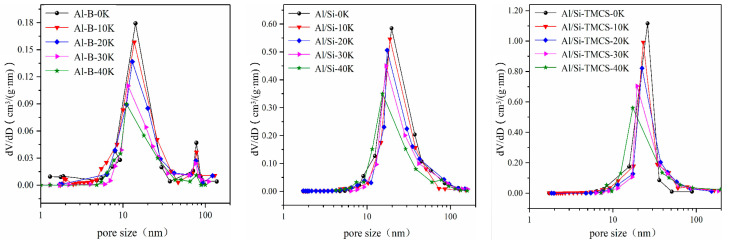
Pore size distribution of the adsorbents after loading.

Affected by the surface –OH, the impregnation process greatly reduces the specific surface area (from 250.65 m^2^/g to 140.49 m^2^/g) and specific pore volume (from 0.49 cm^3^/g to 0.38 cm^3^/g) of Al-B, and the pore size becomes smaller, indicating that the impregnation process causes the Al-B framework to shrink. However, the specific surface area (from 635.32 m^2^/g to 619.54 m^2^/g) and specific pore volume (from 2.43 cm^3^/g to 2.39 cm^3^/g) of Al/Si-TMCS almost do not change, indicating that it maintains a good structure during the impregnation process. With the increase of the loading, the specific surface area, specific pore volume, and the most probable pore size of Al aerogels under different modification conditions are continuously decreasing, indicating that more and more K_2_CO_3_ is filled into the pores, and the change in the content of K in the adsorbents can verify this result. As the theoretical loading increases, the actual K_2_CO_3_ content in the adsorbent gradually deviates from its theoretical value, indicating that the loading characteristics gradually deteriorate, during the screening process after equal volume impregnation, unloaded K_2_CO_3_ powder is lost. After loading, the overall pore size distribution of the adsorbents does not change significantly (as shown in Figure 4). The adsorbents are still mesoporous materials, but their most probable pore size has a tendency to become smaller, and their corresponding peak value decreases, which is caused by K_2_CO_3_ entering the hole.

**Figure 5:**
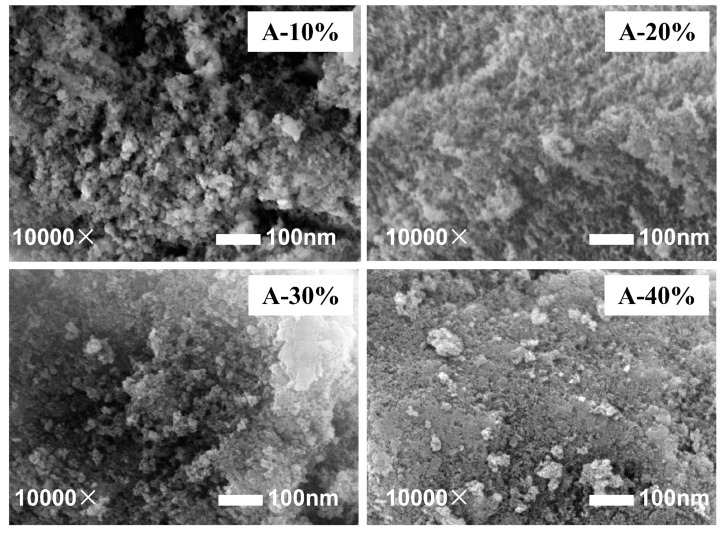

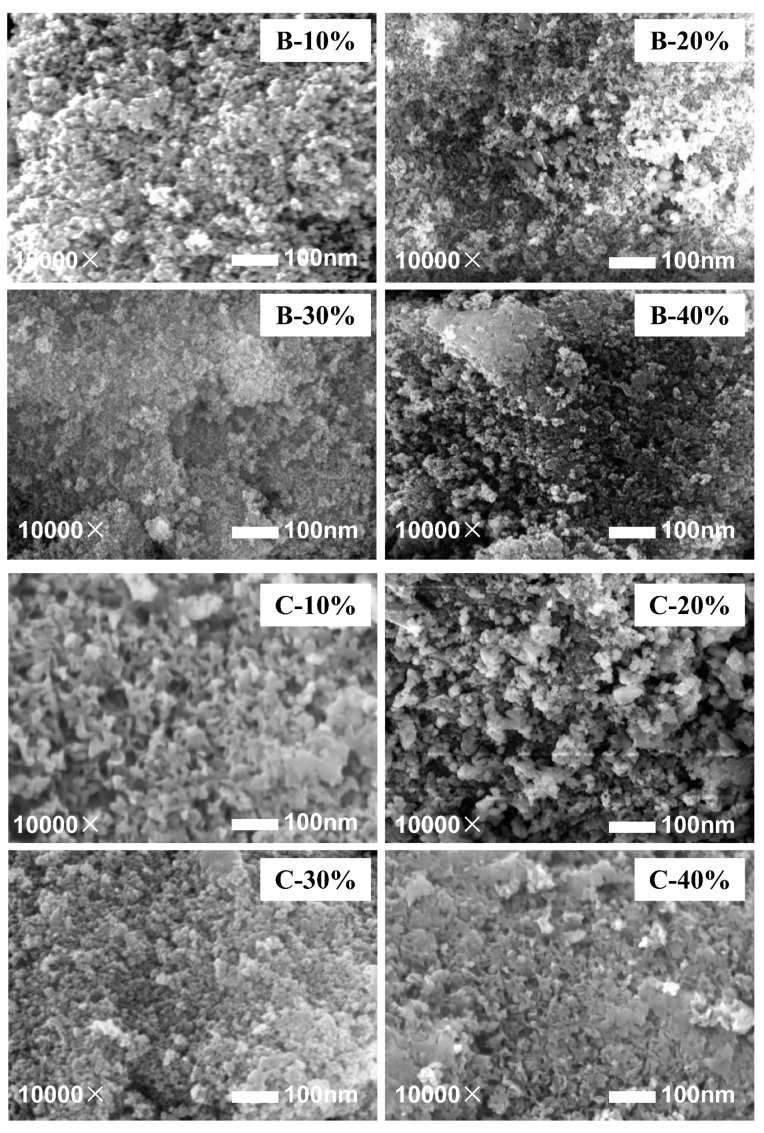
Microstructures of three types of Al aerogels under different loadings. (**A**) Al-B, (**B**) Al/Si, (**C**) Al/Si-TMCS.

In order to study the distribution characteristics of K_2_CO_3_ on different supports, the morphology of the particles was observed using a scanning electron microscope, as shown in Figure 5. In the low loading range, the loose porous structure of the adsorbent can be clearly observed, thus the loading of K_2_CO_3_ do not have a major impact on the microscopic properties of the supports. As the loading increases, more and more K_2_CO_3_ enters the pores of the supports, resulting in the surfaces of the adsorbents gradually becoming dense, especially when the theoretical loading reaches 40%. It can be inferred that K_2_CO_3_ has accumulated on the surface in this case, and the microstructure of the adsorbent deteriorated.

### 3.3. CO_2_ Adsorption Performance

**Figure 6:**
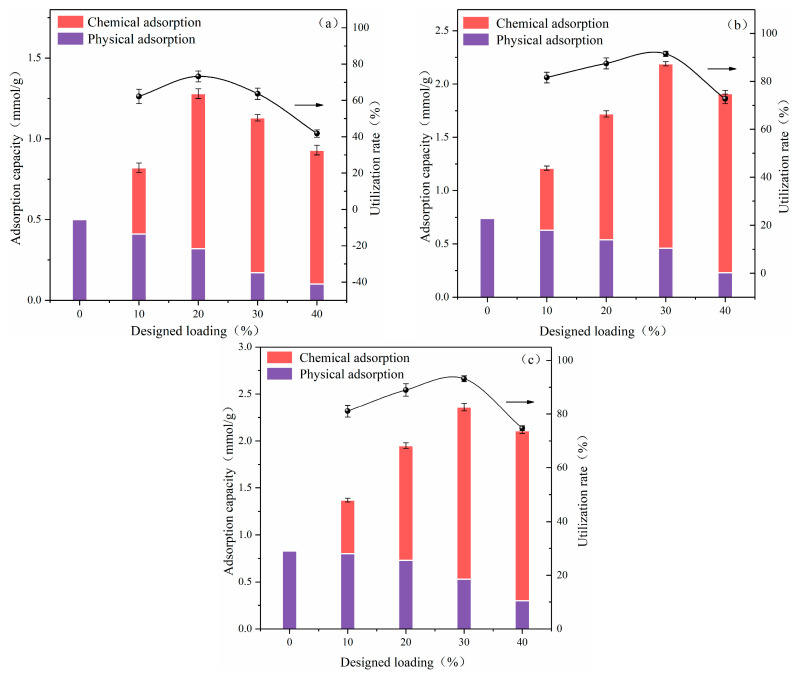
Adsorption capacity of K_2_CO_3_-based adsorbent under 80% N_2_ + 10% CO_2_ + 10% H_2_O (**a**) Al-B, (**b**) Al/Si, (**c**) Al/Si-TMCS.

In the presence of water vapor, the potassium-based adsorbent can increase its CO_2_ adsorption capacity and selectivity through carbonation. Therefore, the carbonation reaction performance of the adsorbents was studied at 70 °C in 80% N_2_ + 10% CO_2_ + 10% H_2_O. For K_2_CO_3_ impregnated Al aerogel, its adsorption of CO_2_ includes both physical and chemical adsorption, and distinguishing these two types of adsorption is important for the study of adsorption mechanisms. In this work, Hsu’s method was used to estimate the physical adsorption capacities of the adsorbents [50]. The specific method is: after the adsorption was saturated, at ambient temperature, the intake gas flow was switched to pure N_2_ at 1 L/min, and the vacuum pump connected to the outlet of the quartz tube was turned on (the vacuum was −0.6 bar). When the CO_2_ concentration of the outlet of the quartz tube was 0, it could be considered that all physically adsorbed CO_2_ had been released, the mass difference of the adsorbent before and after desorption was regarded as the physical adsorption capacity of CO_2_. Figure 6 shows the CO_2_ adsorption capacities and the calculated utilization rates of K_2_CO_3_ with different loadings of adsorbents.

The physical adsorption amounts of Al-B, Al/Si and Al/Si-TMCS to CO_2_ are 0.49, 0.74 and 0.83 mmol/g, respectively, which shows a positive correlation with specific surface area. This is because a large specific surface area can not only increase the contact area between CO_2_ and the adsorbent, but also bring a large number of surface unsaturated adsorption sites. For Al-B-0K, its specific surface area (140.49 m^2^/g) is about 1/3 of Al/Si-0K (446.22 m^2^/g), but the physical adsorption is not much different from that of Al/Si, which may be related to the more –OH on the surface of Al-B. Deng found that –OH can promote the adsorption of CO_2_ [51].

Except for 10%, the adsorption processes of CO_2_ by adsorbents under different loadings are mainly chemical adsorption. As the loading increases, the contribution of physical adsorption to the entire adsorption process decreases. The physical adsorption capacity of the adsorbents are the smallest when the design loading reaches 40%. This is mainly due to the increasing amount of K_2_CO_3_ filling the pores of the Al aerogel, resulting in a decrease in the specific surface area and specific pore volume of the adsorbent, which in turn affects the amount of adsorbed CO_2_.

In the presence of water vapor, as the loading increases, there is a peak in the CO_2_ adsorption capacity result of each type of adsorbent. In the lower loading range, the increase of the loading brings more active sites, which leads to an increase in the amount of adsorption [52]. When the loading is too large, the active components cannot be dispersed uniformly and accumulate. For one thing, the increase of particles leads to a decrease in activity, and for another, the pores are blocked and the resistance to mass transfer is increased, both of them lead to a decrease in the adsorption capacity. Specifically, the adsorption capacity results of Al/Si and Al/Si-TMCS show a peak when the design loading is 30%, while the peak of Al-B appears at 20%. and the peak adsorption capacities are 2.36, 2.22, and 1.27 mmol/g, respectively, its value is higher than the potassium-based adsorbent shown in Table 1. In industrial applications, high adsorption capacity can reduce the adsorption cost by reducing the amount of adsorbent and the size of the equipment. The difference in the positions of the peaks of the three types of adsorbents is due to the different loading characteristics of the three supports for K_2_CO_3_. Al-TMCS and Al-TEOS have a relatively ordered single mesoporous structure, and the specific surface area and specific pore volume are sufficiently large, so that K_2_CO_3_ has a larger dispersion threshold on the surface; while the specific surface area and specific pore volume of Al-B are smaller, and it has a wide pore size distribution with an ink bottle-like pore structure, these factors cause K_2_CO_3_ to reach the dispersion threshold and start enrichment at a lower loading, resulting in increased grain size and increased mass transfer resistance, which in turn reduces its activity during the carbonation reaction. The utilization rate of K_2_CO_3_ has the same trend as the amount of CO_2_ adsorption, which can also be explained by the above mechanism. The maximum K_2_CO_3_ utilization rates of the three types of adsorbents are 74.1%, 91.1%, and 93.2%, respectively. This difference may be due to the different particle sizes of K_2_CO_3_ on the surface and the different optimal carbonation reaction temperatures caused by different activities of different particle sizes of K_2_CO_3_ [53].

### 3.4. Adsorption Mechanism

**Figure 7:**
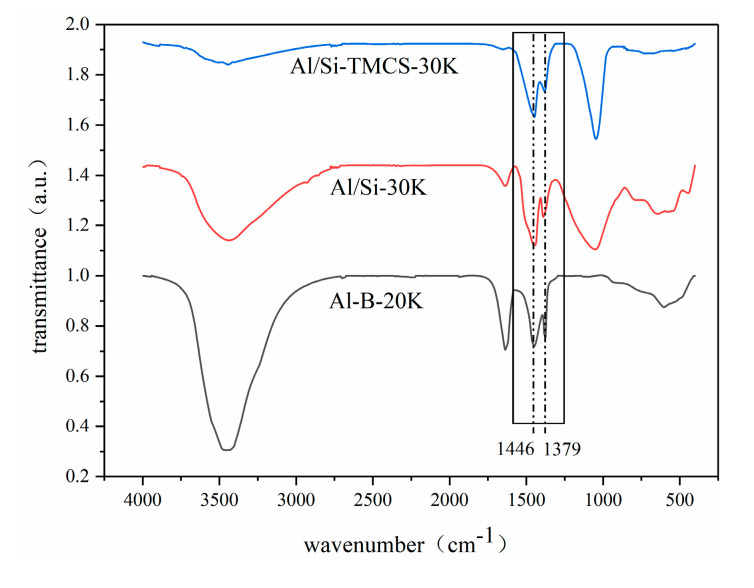
FT-IR spectra of the adsorbents after loading.

**Figure 8:**
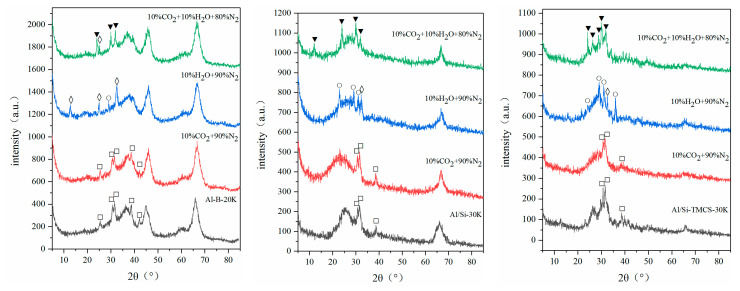
XRD spectra of K_2_CO_3_-based adsorbent in different atmospheres. ☐—K_2_CO_3_, ◊—K_2_CO_3_∙1.5 H_2_O, ο—K_4_H_2_(CO_3_)_3_∙1.5 H_2_O, ▼—KHCO_3_.

In order to obtain the CO_2_ adsorption mechanism of K_2_CO_3_-based Al aerogels, FT-IR analysis was carried out on Al-B-20K, Al/Si-30K and Al/Si-TMCS-30K, and XRD analysis was carried out on Al-B-20K, Al/Si-30K and Al/Si-TMCS-30K adsorbed in 10% CO_2_ + 90%N_2_, 10% H_2_O + 90% N_2_ and 10% H_2_O + 10% CO_2_ + 80% N_2_ atmosphere, respectively, as shown in Figure 7 and Figure 8.

After loading, peaks related to K_2_CO_3_ (1446 cm^−1^ and 1379 cm^−1^) appear in the FT-IR spectra, other surface functional groups do not change significantly, indicating that K_2_CO_3_ does not affect the surface properties of the supports. According to XRD, only K_2_CO_3_ peaks appear in the adsorbent after loading, and the peak shape of the supports do not change significantly. This shows that K_2_CO_3_ exists on the surface of the support by physical action, no chemical reaction occurs, and the skeleton of the support does not change. After adsorption in 10% CO_2_ + 90% N_2_, the composition of the three samples do not change, indicating that the adsorbent adsorbs CO_2_ by physical action under this condition. After being adsorbed in 10% H_2_O + 90% N_2_ atmosphere, K species mainly exist as K_2_CO_3_∙1.5 H_2_O and K_4_H_2_(CO_3_)_3_∙1.5 H_2_O on Al aerogels, this is consistent with Guo’s research [54], in particular, the peak of K_2_CO_3_∙1.5 H_2_O is stronger in Al-B-20K, which is mainly due to the strong hydrophilicity of Al-B-20K. In 10% H_2_O + 10% CO_2_ + 80% N_2_, K species are mainly KHCO_3_ after adsorption, indicating that most of the K_2_CO_3_ undergoes carbonation to generate KHCO_3_, and K_2_CO_3_∙1.5 H_2_O is also detected in Al-B-20K.

The adsorption process of CO_2_ by K_2_CO_3_-based Al aerogel includes the following steps: (1) H_2_O and CO_2_ diffuse to the outer surface of the adsorbent; (2) H_2_O and CO_2_ diffuse to the inner surface of the adsorbent through the channel; (3) H_2_O and CO_2_ are adsorbed to the solid surface; (4) H_2_O and CO_2_ react with the active center. In this paper, Al-B-20K has a small specific surface area and specific pore volume with an ink bottle-shaped pore, which hinders the internal diffusion process of H_2_O and CO_2_, and the KHCO_3_ product layer is easy to block the pores, these factors are not conducive to the occurrence of a carbonation reaction. In addition, scholars generally believe that there are two possible intermediate products of K_2_CO_3_ carbonation reaction: K_2_CO_3_∙1.5 H_2_O and K_4_H_2_(CO_3_)_3_∙1.5 H_2_O, and the surface properties of the support and the distribution characteristics of K_2_CO_3_ will affect the formation of intermediate products during the carbonation reaction [53,54,55,56,57]. Al-B-20K has strong hydrophilicity, under a watery atmosphere, K_2_CO_3_ will react with H_2_O to generate a large amount of K_2_CO_3_∙1.5 H_2_O. In terms of crystal structure, compared with K_4_H_2_(CO_3_)_3_∙1.5 H_2_O, K_2_CO_3_∙1.5 H_2_O is more difficult to achieve the conversion to KHCO_3_, and supports with better microscopic properties can promote this conversion [54].However, due to the small specific surface area and specific pore volume of Al-B, the dispersibility of formed K_2_CO_3_∙1.5 H_2_O is poor, which makes it more difficult to convert into KHCO_3_, resulting in the presence of K_2_CO_3_∙1.5 H_2_O in Al-B-20K after adsorption in 10% H_2_O + 10% CO_2_ + 80% N_2_, which not only affect the conversion rate of carbonation, but also cause valueless consumption of energy in the regeneration process due to the decomposition of K_2_CO_3_∙1.5 H_2_O. The Al/Si-30K and Al/Si-TMCS-30K have a large specific surface area and well-developed pore structure, which provides a suitable channel and place for the diffusion and reaction of H_2_O and CO_2_. At the same time, due to the well-developed pore structure, the heat generated inside the adsorbent during the carbonation reaction can be taken away in time, thereby avoiding local over-temperature of the adsorbent and affecting the carbonation performance. Therefore, compared with Al-B, Al/Si and Al/Si-TMCS are more suitable as K_2_CO_3_ supports for CO_2_ capture.

### 3.5. Adsorption Kinetics

**Figure 9:**
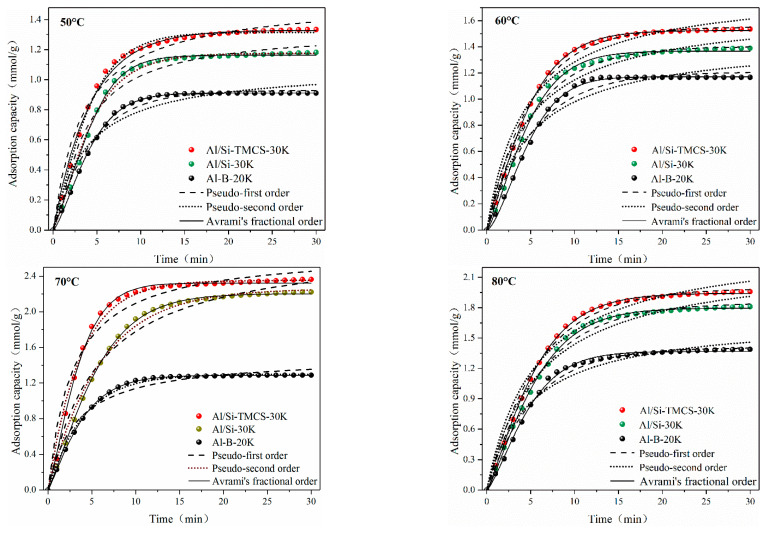
Adsorption kinetics fitting results of adsorbents at different temperatures.

Temperature is an important factor that affects the carbonation reaction. In this paper, the CO_2_ adsorption kinetics of Al-B-20K, Al/Si-30K and Al/Si-TMCS-30K in the range of 50–80 °C were studied. The experimental results were fitted using pseudo-first order, pseudo-second order, and Avrami’s fractional order models. The expressions of the three models are shown in Equations (5)–(7), respectively [58,59,60]. The cumulative adsorption curve and fitting results at different temperatures are shown in Figure 9, and the obtained kinetic parameters are listed in Table 4.

(5) $$q_{t}\;{=\;q}_{e}\left[1\;-\;\exp\left(-k_{1}t\right)\right]$$

where q_t_ is adsorption capacity per unit mass of adsorbent at time t, g/g; q_e_ is adsorption capacity per unit mass of adsorbent at equilibrium, g/g; t is the adsorption time, min; k_1_ is the pseudo-first order rate Constant, min^−1^.

(6) $$q_{t}\;=\;\frac{q_{e}^{2}k_{2}t}{{1\;+\;q}_{e}k_{2}t}$$

where k_2_ is the pseudo-second order rate constant, gmmol^−1^min^−1^.

(7) $$q_{t}{\;=\;q}_{e}\left[1\;-\;\exp{\left(-k_{A}t\right)}^{n_{A}}\right]$$

where k_A_ is the Avrami kinetic rate constant, min^−1^; n_A_ is the Avrami index, when n_A_ = 2, the reaction conforms to a one-dimensional growth mechanism; when n_A_ = 3, the reaction conforms to a two-dimensional growth mechanism.

In the range of 50–80 °C, the adsorption capacity of Al-B-20K shows a gradual increase trend with the increase of temperature, and it reached a maximum of 1.37 mmol/g at 80 °C. However, the adsorption capacities of Al/Si-30K and Al/Si-TMCS-30K gradually increase with the increase of temperature in the low temperature region, and reach a peak when the temperature reaches 70 °C, and then with the rise in temperature, the adsorption capacities have a certain reduction. In the process of CO_2_ adsorption by the adsorbents, physical adsorption and chemical adsorption coexist, and temperature has an important influence on both. In the low temperature region, the increase in temperature is conducive to increasing the activity of the adsorbent, making it easier for the chemical bonds of the active components to be cleaved, which in turn leads to a more prone carbonation reaction. However, when the temperature is too high on the one hand, the increase in temperature is not conducive to the physical adsorption of CO_2_ by the adsorbent; on the other hand, an excessively high temperature exacerbates the exothermic effect of the carbonation reaction, which leads to the shift of equilibrium to desorption. For Al/Si-30K and Al/Si-TMCS-30K, due to their better micro characteristics, thermodynamics play a more important role in the process of CO_2_ adsorption than kinetics; while Al-B-20K has a poor pore structure, and K_2_CO_3_ exists as larger particles with a higher crystallinity, which results in higher energy barriers for its active components, and so higher temperature are required to excite its activity.

As can be seen from Figure 9, compared to the pseudo-first order and pseudo-second order models, Avrami’s fractional order can better describe the adsorption processes of three adsorbents on CO_2_, while the data fitted by the pseudo-first order model shows the largest deviation from the actual data.

In general, the pseudo-first order model is generally suitable for describing the pure physical adsorption of adsorbents at a quite low surface coverage, and regardless of the role of chemical bonds, while the pseudo-second order model is mainly used to describe pure chemical adsorption. At 50 °C, the pseudo-first order model fits Al-B-20K better than the pseudo-second order model, indicating that at this temperature, the carbonation activity of Al-B-20K is not strong, and the physical adsorption of CO_2_ by the adsorbent is dominant. As the temperature increases, the correlation coefficient (R^2^) obtained by the pseudo-second order model is gradually greater than that of the pseudo-first order model, indicating that the increase of temperature weakens the influence of external mass transfer on the CO_2_ adsorption process, and chemical adsorption gradually becomes the speed control step of the entire adsorption process.

In the range of 50–80 °C, the correlation coefficients of pseudo-second order model of Al/Si-30K and Al/Si-TMCS-30K are always larger than pseudo-first order model, which indicates that chemical adsorption always dominates the entire adsorption process. At the same time, as the temperature increases, the influence of chemical adsorption on the entire adsorption process becomes more intense.

Avrami’s fractional order model is based on the mechanism of particle nucleation and crystal growth, which is mostly used to explain the complex adsorption mechanism of adsorbate on adsorbent. Among the three models, Avrami’s fractional order shows the best fit to the three adsorbents, indicating that the adsorption of CO_2_ by the adsorbent is a complex process involving both physical and chemical interactions. It can be seen from the kinetic parameters obtained from the Avrami’s fractional order model that as the adsorption temperature increases, the adsorption rate shows a gradually increasing trend. The adsorption rate constant of Al-B-20K is significantly lower than that of the other two adsorbents, which is caused by the poor microstructure of the support and insufficient K_2_CO_3_ activity on the support surface. At the same time, as the temperature increases, the n_A_ values are all around 2, indicating that the increase in temperature has not changed the adsorption mechanism of CO_2_ on the adsorbent, and the adsorption of CO_2_ on the adsorption site conforms to the one-dimensional growth mechanism.

**Figure 10:**
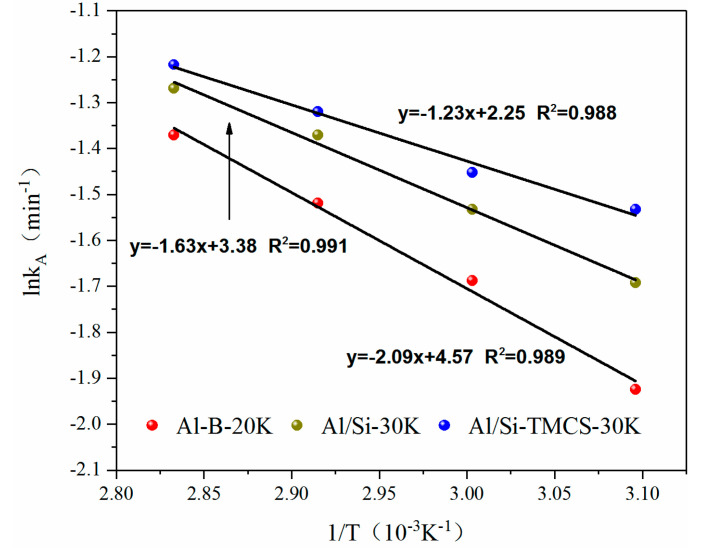
Arrhenius plot of k_A_ obtained by Avrami’s fractional order.

Among the three adsorption kinetic models, Avrami’s fractional order model showed the best fitting result, so the Arrhenius equation was used to fit the kinetic parameter k_A_ to obtain the apparent activation energy of the adsorption process, and the results are shown in Figure 10.

The apparent activation energy of the three adsorbents are 17.38 kJ/mol, 13.55 kJ/mol, and 10.22 kJ/mol, respectively. Compared with Al/Si-30K and Al/Si-TMCS-30K, Al-B-20K shows a higher activation energy, indicating that Al-B-20K requires more energy in the process of CO_2_ adsorption, which verifies the above result.

### 3.6. Cycle Performance of Al/Si-30K and Al/Si-TMCS-30K

In order to have practical value in industrial applications, the CO_2_ adsorbent must also have a stable regeneration capacity. In this work, 15 adsorption-regeneration experiments were performed on Al/Si-30K and Al/Si-TMCS-30K, regeneration experiments were carried out at 150 °C in a pure nitrogen atmosphere. The failure mechanisms of the adsorbents were studied through the microstructural changes of the adsorbents during cycling.

**Figure 11:**
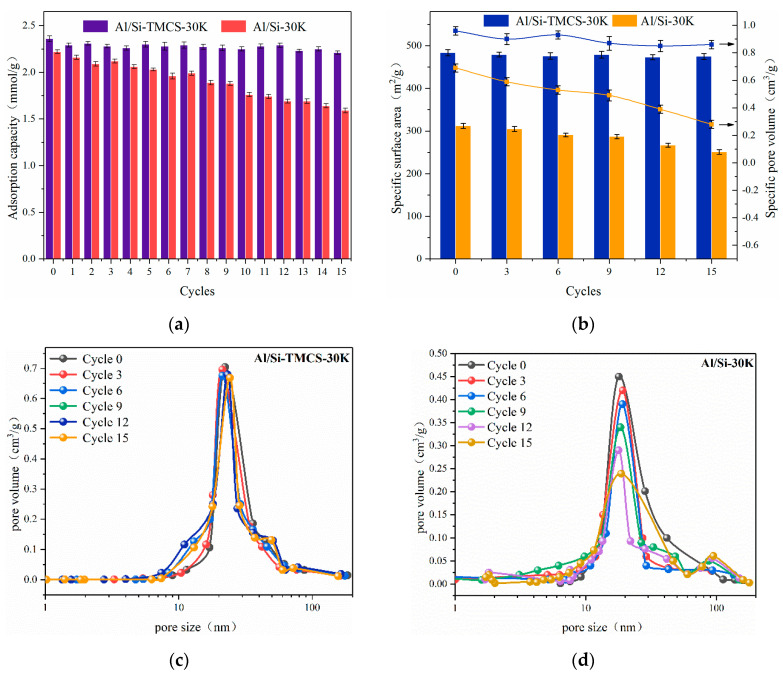
Cycling performance of Al-TMCS-30K and Al-TEOS-30K. (**a**) Adsorption capacity during the cycle. (**b**) Microstructural parameters during the cycle. (**c**) Pore size distribution during the cycle. (**d**) Pore size distribution during the cycle.

The cycling performance of Al-TMCS-30K during the 15 cycles are shown in Figure 11. As the number of cycles increased, the CO_2_ adsorption capacity of Al/Si-TMCS-30K showed a slight decrease. After 15 adsorption-regeneration cycles, the adsorption capacity is reduced from only 2.36 mmol/g to 2.21 mmol/g, indicating that it has a good cycle performance, while the reduction of Al/Si-30K reaches 28.4%. During 15 cycles, the microstructure of Al/Si-TMCS-30K has not changed much, while the specific surface area and specific pore volume of Al/Si-30K have decreased to a large extent (as shown in Figure 11b). At the same time, the pore size distribution of Al/Si-30K has changed greatly, and new micropores and macropores have been generated (as shown in Figure 11d). It is speculated that the decrease in the adsorption performance of Al/Si-30K is mainly due to the destruction of the microstructure of the support during the cycles. Since the adsorbents adsorb CO_2_ in the water vapor atmosphere, and Al/Si-30K has a certain hydrophilicity, its structure is easily damaged during the continuous water absorption-drying process. On the one hand, the destruction of the support skeleton adversely affects the adsorption and diffusion of CO_2_, on the other hand, the collapsed skeleton may wrap K_2_CO_3_, which hinders the carbonation reaction. The hydrophilicity of Al/Si-TMCS-30K is greatly reduced by TMCS modification, it maintains a good structure in the cycle, so it has a good cycle performance. 

## 4. Conclusions

Aiming at the removal of CO_2_ from coal-fired flue gas, a CO_2_ adsorbent with Al aerogel used as the support and K_2_CO_3_ used as the active component was proposed. The effects of surface modifiers and K_2_CO_3_ loadings on the adsorption characteristics were studied. The main conclusions obtained are as follows:

(1) The modification of TMCS and TEOS during the preparation process can effectively reduce the shrinkage and collapse of the Al aerogel structure by ambient pressure drying, so that it maintains a good microstructure.

(2) TEOS + TMCS-modified Al_2_O_3_ aerogel shows a best CO_2_ adsorption performance at 30% K_2_CO_3_ loading (Al/Si-TMCS-30K), and the CO_2_ adsorption capacity and K_2_CO_3_ utilization rate are 2.36 mmol/g and 93.2%, respectively.

(3) Avrami’s fractional order kinetic model can well fit the CO_2_ adsorption process of potassium-based adsorbents, which proves that the adsorption processes of CO_2_ by the adsorbents are a complex adsorption mechanism including physical and chemical adsorption. At the same time, from the point of view of adsorption rate constant, compared with common potassium-based adsorbents, Al/Si-TMCS-30K has a faster adsorption kinetics. In the range of 50–80 °C, as the temperature increases, the influence of chemical adsorption on the entire adsorption process increases.

(4) After 15 adsorption-regeneration cycles, the CO_2_ adsorption capacity of Al/Si-TMCS-30K remains basically stable, and the microstructure do not change much. 

(5) In view of the high CO_2_ adsorption capacity, fast adsorption kinetics, and stable regeneration performance of Al/Si-TMCS-30K, it has a great application potential in the field of capturing CO_2_ in coal-fired flue gas.

## Figures and Tables

**Table 1:** A summary of adsorption capacity of potassium-based adsorbents.

Adsorbents	Loading (%)	Reactor	Reaction Conditions	CO_2_ Capture Capacity (mmol/g)	References
K_2_CO_3_/AC	12.5	Fixed bed	0.5% CO_2_ + 1.8% H_2_O 20 °C	0.87	[26]
K_2_CO_3_/Al_2_O_3_	28.5	Fixed bed	0.5% CO_2_ + 1.8% H_2_O 20 °C	1.18	[26]
K_2_CO_3_/5A	11.2	Fixed bed	0.5% CO_2_ + 1.8% H_2_O 20 °C	0.34	[26]
K_2_CO_3_/13X	16.2	Fixed bed	0.5% CO_2_ + 1.8% H_2_O 20 °C	0.53	[26]
K_2_CO_3_/SG	37.5	Fixed bed	0.5% CO_2_ + 1.8% H_2_O 20 °C	0.15	[26]
K_2_CO_3_/AC	15.0	Fixed bed	1% CO_2_ + 2% H_2_O 60 °C	1.01	[27]
K_2_CO_3_/AC	33.0	Fixed bed	1% CO_2_ + 9% H_2_O 60 °C	2.09	[28]
K_2_CO_3_/AC	24.0	Thermogravimetric Analysis (TGA)	15% CO_2_ + 15% H_2_O 60 °C	1.67	[29]
K_2_CO_3_/Al_2_O_3_	25.0	Bubbling fluidized-bed	13% CO_2_ + 13% H_2_O 60 °C	1.66	[30]
K_2_CO_3_/Al_2_O_3_	35.0	Fluidized bed	13% CO_2_ + 9% H_2_O 60 °C	1.48	[31]
K_2_CO_3_/Al_2_O_3_	30.0	Fixed bed	1% CO_2_ + 9 % H_2_O 60 °C	1.93	[32]
K_2_CO_3_/SG	20.5	TGA	1% CO_2_ + 2% H_2_O 20 °C	0.53	[33]
K_2_CO_3_/SiO_2_	20.0	Fixed bed	1% CO_2_ + 2% H_2_O 20 °C	1.34	[34]
K_2_CO_3_/FeOOH	33.0	Fixed bed	1% CO_2_ + 2% H_2_O 60 °C	1.12	[35]

**Table 2:** Microstructure parameters of the aerogels.

Adsorbents	Specific Surface Area (cm^2^/g)	Specific Pore Volume (cm^3^/g)	Most Probable Pore Size (nm)
Al-B	250.65 ± 3.21	0.49 ± 0.02	15.6 ± 0.1
Al/Si	496.22 ± 5.43	1.59 ± 0.04	20.4 ± 0.2
Al/Si-TMCS	635.32 ± 5.57	2.43 ± 0.11	26.4 ± 0.2

**Table 3:** Microstructure parameters of the adsorbents.

Adsorbents	Specific Surface Area (cm^2^/g)	Specific Pore Volume (cm^3^/g)	Most Probable Pore Size (nm)	Actual Loading (%)
Al-B-0K	140.49 ± 1.87	0.38 ± 0.01	14.1 ± 0.1	0
Al-B-10K	105.41 ± 1.94	0.28 ± 0.01	13.5 ± 0.1	9.1 ± 0.2
Al-B-20K	70.36 ± 1.10	0.15 ± 0.00	12.9 ± 0.1	18.1 ± 0.3
Al-B-30K	40.53 ±1.03	0.09 ± 0.00	10.9 ± 0.1	20.8 ± 0.3
Al-B-40K	10.56 ± 0.86	0.02 ± 0.00	10.1 ± 0.1	27.4 ± 0.4
Al/Si-0K	446.22 ± 4.96	1.39 ± 0.03	19.7 ± 0.2	0
Al/Si-10K	403.26 ± 3.99	1.13 ± 0.02	19.0 ± 0.2	9.8 ± 0.1
Al/Si-20K	353.64 ± 3.64	0.87 ± 0.01	17.8 ± 0.1	18.6 ± 0.1
Al/Si-30K	312.21 ± 3.42	0.69 ± 0.01	16.9 ± 0.2	26.1 ± 0.2
Al/Si-40K	270.55 ± 2.32	0.24 ± 0.01	15.3 ± 0.1	31.9 ± 0.2
Al/Si-TMCS-0K	619.54 ± 5.65	2.39 ± 0.04	26.0 ± 0.2	0
Al/Si-TMCS-10K	578.75 ± 5.12	2.01 ± 0.04	24.1 ± 0.2	9.7 ± 0.1
Al/Si-TMCS-20K	526.74 ± 4.86	1.53 ± 0.02	22.4 ± 0.1	18.9 ± 0.1
Al/Si-TMCS-30K	483.41 ± 4.66	0.96 ± 0.01	19.2 ± 0.1	27.1 ± 0.3
Al/Si-TMCS-40K	420.83 ± 4.21	0.41 ± 0.01	17.8 ± 0.1	33.4 ± 0.2

**Table 4:** The kinetic parameters obtained by different adsorption kinetic models.

Adsorbents	Temperature (°C)	Adsorption Capacity (mmol/g)	Pseudo-First Order			Pseudo-Second Order			Avrami’s Fractional Order			
			R^2^	k_1_	q_e_	R^2^	k_2_	q_e_	R^2^	k_A_	q_e_	n_A_
Al-B-20K	50	0.91	0.982	0.072	0.93	0.952	0.161	1.06	0.988	0.146	0.87	1.996
	60	1.18	0.979	0.104	1.29	0.967	0.174	1.22	0.991	0.185	1.21	1.984
	70	1.27	0.973	0.135	1.49	0.973	0.185	1.38	0.993	0.219	1.31	2.007
	80	1.37	0.969	0.158	1.46	0.981	0.198	1.43	0.996	0.254	1.39	1.995
Al/Si-30K	50	1.18	0.978	0.154	1.34	0.981	0.162	1.17	0.993	0.184	1.16	1.984
	60	1.39	0.973	0.162	1.60	0.982	0.178	1.38	0.994	0.216	1.36	1.989
	70	2.22	0.969	0.173	2.26	0.985	0.181	2.26	0.995	0.254	2.20	1.996
	80	1.81	0.960	0.1836	2.22	0.985	0.189	1.83	0.994	0.281	1.79	2.011
Al/Si-TMCS-30K	50	1.33	0.973	0.164	1.54	0.979	0.171	1.32	0.993	0.216	1.31	1.985
	60	1.53	0.970	0.176	1.84	0.982	0.185	1.55	0.995	0.234	1.52	1.996
	70	2.36	0.968	0.189	2.45	0.983	0.194	2.32	0.998	0.267	2.35	1.994
	80	1.95	0.963	0.192	2.43	0.984	0.199	1.99	0.998	0.296	1.94	2.001

## Abbreviation

TEOS	Tetraethyl orthosilicate
TMCS	Trimethyl chlorosilane
Al-B-20K	Al-B with 20 wt.% K_2_CO_3_
Al/Si-30K	Al/Si with 30 wt.% K_2_CO_3_
Al/Si-TMCS-30K	Al/Si-TMCS with 30 wt.% K_2_CO_3_
